# HPV vaccine: uptake and understanding among global Indigenous communities – a qualitative systematic review

**DOI:** 10.1186/s12889-021-12147-z

**Published:** 2021-11-10

**Authors:** Brianna Poirier, Sneha Sethi, Gail Garvey, Joanne Hedges, Karen Canfell, Megan Smith, Xiangqun Ju, Lisa Jamieson

**Affiliations:** 1 Australian Research Centre for Population Oral Health, University of Adelaide, 4 North Terrace, 4 North Terrace, Adelaide, SA 5000 Australia; 2 School of Public Health, Faculty of Medicine, University of Queensland, St Lucia, QLD 4072 Australia; 3 The Daffodil Centre at the University of Sydney, A Joint Venture with Cancer Council, PO Box 572, Kings Cross, NSW 1340 Australia

**Keywords:** Indigenous women health, Human papillomavirus infections, Cervical cancer, Qualitative systematic review, HPV vaccine

## Abstract

**Background:**

Indigenous populations have a high prevalence of Human Papillomavirus (HPV) infection and a high incidence of HPV associated cancers, such as cervical and oropharyngeal cancer. There is an effective HPV vaccination program in almost all developed countries to prevent the incidence of cervical cancer, but reports suggest that the uptake of these vaccinations by Indigenous populations is low. The objective of this qualitative systematic review was to explore the knowledge and beliefs of global Indigenous populations regarding HPV vaccines. This review was performed to identify the barriers faced by Indigenous peoples and to provide evidence for more effective and acceptable execution of vaccination policies for Indigenous peoples.

**Methods:**

Two investigators independently searched MEDLINE, PubMed, SCOPUS, and Web of Science databases using a pre-specified search strategy to identify qualitative studies on narratives of Indigenous peoples regarding HPV vaccine awareness, knowledge and experiences across all geographic and income-level settings.

**Results:**

After performing the literature search and quality appraisals 5 papers were included in the final review. Three core synthesised findings were identified: reasons for acceptance or hesitancy, and areas for improvement. Lack of correct knowledge and mistrust in the healthcare system were important categories observed in all papers included in the review. Other categories within the conceptual model included prioritising disease prevention, health professional guidance, family support and supportive community environment.

**Conclusion:**

Qualitative systematic reviews are an excellent means of exploring the gaps in current healthcare practices. Indigenous healthcare research should be grounded in community experiences and feedback. This review provides insights into HPV vaccination understanding and acceptance amongst Indigenous populations, from which recommendations for increasing resonance of vaccination strategies with Indigenous communities can be formed.

**Supplementary Information:**

The online version contains supplementary material available at 10.1186/s12889-021-12147-z.

## Background

Humans have highly evolved immune systems, which possess considerable abilities to recognise, remember, and fight pathogens. The technology of vaccines capitalises on this ability and induces an immune response that confers protection against infection and/or disease on subsequent exposure to a pathogen [1]. This has led to a significant decline in the spread of highly infectious diseases and has provided opportunities to eradicate diseases such as polio and smallpox.

Human Papillomavirus (HPV), with more than 200 types, is one of the most commonly sexually transmitted infections and is associated with cancers of the cervix, anus, and oropharynx [2–4]. HPV infects 80% [5] of people at least once in their lifetime, but due to its rapid clearance rate, many people never become aware of infection. Malignant changes can be anticipated if one of the 14 high-risk HPV types are persistent in the human body for a prolonged period [6, 7].

The three most widely available vaccines against HPV infection are Gardasil (Quadrivalent; Merck & Co., Kenilworth, NJ, USA), Gardasil9 (Nonavalent; Merck & Co., Kenilworth, NJ, USA), and Cervarix (Bivalent; GSK, Brentford, UK) [8]. Although preventive vaccines have been available for women since 2006 [9], statistics demonstrate a sharp increase in the number of reported HPV-related oropharyngeal, anal, and penile lesions cases among men [9–11]. Although many countries introduced a vaccination program for men after observing the increased incidence of HPV-related cancers, it is important to note that the impact of HPV vaccination on HPV-related oropharyngeal, anal, and penile lesions in men will not be immediately observed due to the natural progression of HPV infection and the variations of clearance or persistence [12]. A systematic review in 2016 demonstrated that while substantial proportions of women from high- and middle-income countries were being vaccinated, women in low-income countries or regions, who are potentially at a higher risk, had difficulties accessing vaccinations [13, 14]. Studies demonstrate growing inequalities in both the distribution and uptake of vaccines [15–17]. Steps are being taken to address circumstances of inaccessible HPV vaccination programs, with considerable efforts being made to create awareness and to make vaccines more widely available for men and those in disadvantaged communities [15–17].

Following the 2018 Call to Action [18], the World Health Organisation (WHO) launched a cervical cancer elimination strategy in 2020, with three main objectives of preventing, screening, and treating HPV associated cervical cancers. The targets for the WHO strategy include 90% vaccination rates, 70% screening rates and treatment for 90% of the invasive cancers for women in all countries by the year 2030 [14].

Low vaccination rates are related to vaccine hesitancy in addition to vaccine inaccessibility. Hesitancy has been defined as an expression of concern or doubt about the value or safety of vaccination; thus, the concerns are not limited only to those who decline to get vaccinated but who encourage others to decline vaccination [19]. Vaccine decisions are personal and complex. Hesitation for vaccinations can be attributed to a variety of factors, such as safety concerns and incorrect knowledge [20, 21]. Further exploration of hesitancy is needed to identify and better understand hesitations in order to address barriers and improve vaccine uptake. Vaccination attitudes are influenced by people at every level of the healthcare system including healthcare workers, community members, and public health professionals. It has been reported that healthcare professionals have expressed difficulty in building trusting relationships with patients leading to an information deficit [19]. Misinformation and lack of awareness has been described as the most common reasons for developing a hesitant attitude towards vaccinations [19].

Globally, Indigenous peoples bear a high burden of chronic and infectious diseases, especially in developed countries [22]. Indigenous peoples includes all “people with a historical continuity with pre-evasion and pre-colonial societies that developed on their territories, and who consider themselves distinct from other sectors of the societies now prevailing on those territories” [23]. The Centre for Disease Control and Prevention (CDC) in the United States has reported an increased burden of sexually transmitted diseases among Indigenous communities [24]. A higher pooled prevalence of HPV infection in Indigenous populations has been observed [25], compared to the pooled prevalence of general populations [11, 13]. While HPV vaccine coverage has been reported as high, course completion is generally lower for Indigenous adolescents [26]. Trust between Indigenous community members and healthcare workers is central to vaccination strategies. These relationships have significant implications for researchers and policy makers. Increasing vaccination rates requires a coordinated and engaged strategy.

The objective of this qualitative systematic review was to explore the knowledge and beliefs of global Indigenous populations regarding HPV vaccines. This review was performed to identify the barriers faced by Indigenous peoples and to provide evidence for more effective and acceptable execution of vaccination policies for Indigenous peoples.

## Methods

This systematic review has been registered in PROSPERO (CRD42021239160) and the Joanna Briggs Systematic Reviews register. A prior search of the PROSPERO register revealed no similar studies. Both the PRISMA guidelines [27] and the Enhancing Transparency in Reporting the Synthesis of Qualitative Research (ENTREQ) statement (Table 1) were followed.

**Table 1:** ENTREQ Checklist

Item	Description	Reported on Page #
Aim	The objective of this systematic review was to explore the knowledge, beliefs and experiences of Indigenous populations all over the world regarding HPV vaccines.	2
Synthesis methodology	Content analysis guided initial data extraction for synthesis, and the conceptual model provided a theoretical framework to present the synthesised findings	5
Approach to searching	Pre-established search strategy which involved using terms describing the population of interest, the phenomenon we are researching as well as study designs to be included	3
Inclusion criteria	*Inclusion*: The study focused on the knowledge, views, experiences and barriers faced by women and/or health care workers of Indigenous identity regarding HPV vaccinations. Findings contained personal illustrations or first-person accounts of HPV vaccine knowledge and experiences. The study was qualitative or mixed methods (with clear qualitative examples) HPV vaccination was the phenomenon of interest. The study was available in English. The study was available in hardcopy or in downloadable form. The study was published prior to January, 2021 *Exclusion*: Based only on HPV infections and associated cancers. Quantitative only studies	4
Data sources	MEDLINE, PubMed, SCOPUS, and Web of Science databases; each search tailored per the design of individual database. In our search for published studies, we made use of facilities when given to run ‘related’ searches and the bibliography of each article was manually scanned for possible additions to the study	3
Electronic Search Strategy	Terms utilised for literature search included: ‘HPV’ ‘Vaccine’ ‘Indigenous’ ‘narrative’ ‘story’ ‘qualitative’ ‘mixed methods’	3
Study Screening methods	Two independent researchers screened studies for inclusion in the qualitative systematic review. Titles were first reviewed, then abstracts and those considered relevant by either investigator advanced to full text review.	2 and 3
Study characteristic	See Table 3	Table 3
Study selection results	179 records were returned from initial search, 116 were excluded due to duplication, 63 shortlisted, 5 studies fully satisfied inclusion criteria.	Figure 1
Rationale for appraisal	Utilizing JBI SUMARI software, articles were appraised according to the CASP (2013) method of quality appraisal.	S2 and S3
Appraisal Items	See S2 and S3	S2 and S3
Appraisal Process	Appraisal was conducted independently by both reviewers and then findings were discussed, and consensus was required before moving forward.	4
Appraisal Results	All 5 articles were included after the appraisal because they satisfied inclusion criteria of personal illustrations	Table 6
Data extraction	All text under headings “Results” and “Conclusions,” as well as all findings under the heading “Discussion” were analysed. Data was manually extracted with highlighters from printed versions of appraised articles and then imputed into the JBI SUMARI software.	Table 4, Table 3
Software	JBI SUMARI	2
Number of Reviewers	Two reviewers independently reviewed articles and extracted data. Findings were then compared, discussed and compiled.	4
Coding	Data was coded from selected articles, going line by line to search for concepts and considering the author-prescribed themes.	5
Study Comparison	All findings were individually highlighted and written on a white board and then connections were made between findings and categories were created based on similarities within and across extracted data.	5
Derivation of themes	The process of deriving themes was abductive.	5
Quotations	Table 4	Table 4
Synthesis output	Results section and Fig. 2	5–10 and Fig. 2

### Positionality

Recognising that personal experiences and opinions heavily influence research perspectives, it is critical for researchers to self-situate. This review is a result of the desire to prioritize individual voices and stories of Indigenous women and healthcare workers. After hearing first-hand accounts of various health disparities experienced by Indigenous women in South Australia, while conducting field work for a different HPV project, the primary reviewers (B.P and S.S) discussed the importance of the person behind each statistic. A desire to synthesise existing knowledge in HPV vaccine literature was established with the aim to identify future research steps and policy actions. While both are non-Indigenous researchers, B. P has qualitative experience with community-engaged scholarship in the context of Indigenous health in Canada and Australia and S. S is an oral pathologist with experience working with Indigenous populations in Australia. The supporting research team consists of Indigenous and non-Indigenous scholars with vast experience in the realm of Indigenous health research.

### Identifying studies for inclusion

The reviewers used a pre-established search strategy [28]**,** which involved using terms (and their edited variants) describing the population of interest, the phenomenon being researched, as well as the included study designs (Supplementary file 1). Two investigators (B.P and S.S) independently screened the literature for eligible articles using MEDLINE, PubMed, SCOPUS and Web of Science databases from inception until 6th January 2021. For example, the search strategy used for PubMed Database was as follows: First Nation/First Nations/Pacific Islander/Pacific Islanders/Torres Strait Islander/Torres Strait* Islanders/ Aborigin*/ Alaska*/ Aleut*/ Amerind*/ American Indian/ Arctic/ Aymara/ Bushmen/ Chukchi/ Chukotka*/ Circumpolar/ Eskimo*/ Greenland*/ Hmong/ Indian*/ Indigen*/ Inuit*/ Inupiaq/ Inupiat/ Khanty/ Maori*/ Mapuche/ Metis/ Native*/ Navaho*/ Navajo*/ Nenets/ Quechua/ Saami/ Sami/ Samoan*/ Siberia*/ Skolt/ Tribal/ Tribe*/ Xingu*/ Yup’ik/ Yupik/ Zuni/“African continental ancestry group”/"African continental ancestry group”/ “Asian continental ancestry group”/“Health Services, Indigenous”/“Oceanic ancestry group”/"arctic regions”/"ethnic groups”, “HPV”, “Human Papillomavirus”, “Papillomavirus”, “HPV 18”, “HPV*”, “Qualitative”, “awareness”, “barriers”, “HPV vaccine”, “vaccine*”. The search was tailored as per the design of individual databases.

In the search for published studies, the reviewers made use of facilities where the option was given to run ‘related’ searches, where similar studies are automatically identified. The bibliography of each article was scanned manually for possible additions to the search. Titles and abstracts were screened by both reviewers independently to assess eligibility, with those considered relevant by either investigator advancing to a full-text review. The investigator pair fully screened articles to identify studies that fulfilled the following criteria:

- The study focused on Indigenous peoples’ [23] knowledge, views, and experiences of HPV vaccinations.
- Findings contained personal illustrations or first-person accounts of HPV vaccine knowledge and experiences.
- The study was qualitative or mixed methods (with clear qualitative examples)
- HPV vaccination was the outcome of interest
- The publication was available in English
- The publication was available in hardcopy or in downloadable form
- The paper was published prior to 6th January, 2021

Exclusion criteria

- Based only on HPV infections and associated cancers
- Quantitative only studies

Any disagreements between the two reviewers were resolved in consultation with a third reviewer (L.J.). While efforts were made to decrease publication bias, the reviewers recognize that limiting the search to the English language could result in loss of data in other Native languages. Additionally, the inclusion of all grey literature could have provided additional findings for the study and decreased possible impacts of publication bias.

### Critical appraisal

There are various validated tools for appraisal of studies; this review employed the JBI (Joanna Briggs Institute) System for the Unified Management, Assessment and Review of Information (SUMARI) critical appraisal tool (Supplementary file 2). This tool includes questions regarding congruity between research philosophies, methodologies, and analysis as well as findings and researcher positionality.

### Data extraction and synthesis

Data were extracted in two phases. The first phase utilised the JBI data extraction tool for all studies, which includes study characteristics, such as location and main findings. For the second phase, the reviewers comprehensively extracted identified findings from each of the included studies. These findings were uploaded to JBI SUMARI and each reviewer independently scored the findings as “Credible”, “Not Supported” or “Unequivocal”; the score for each finding was based on inter-reviewer agreement. The synthesis of findings was done manually by reviewers, which included writing all findings on a white board and identifying common phrases, themes, and concepts. Common themes were grouped, with connections between other themes explored in the context of the HPV vaccinations in Indigenous communities. These categories were then transferred from the white board to the JBI SUMARI tool, and each individual finding was placed within the appropriate category. Finally, the reviewers placed each category within overarching synthesised findings, which reflected the findings from each included study.

## Results

**Fig. 1:**
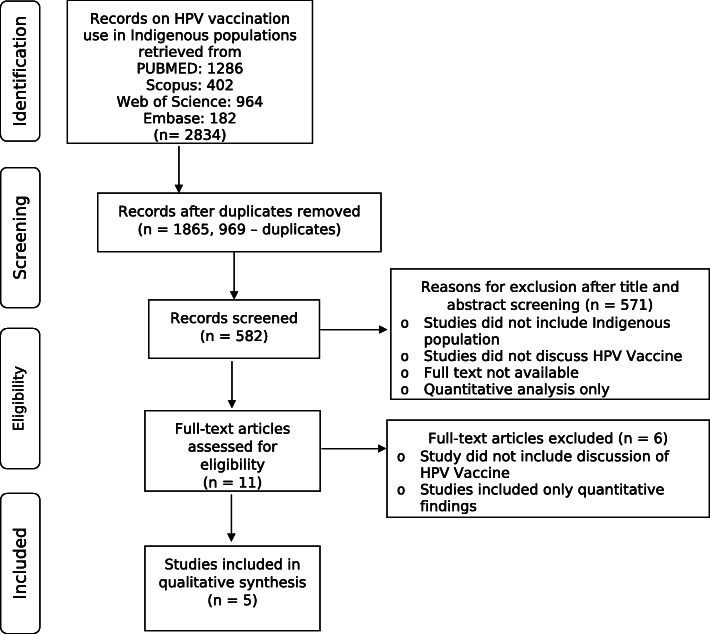
PRISMA flowchart

The literature search returned 2834 records, of which 969 were duplicates, leaving 1865 records after excluding duplicates. After title and abstract screening against established inclusion and exclusion criteria, 11 articles progressed to full-text review. Of the 11 potentially eligible papers, 5 fully satisfied the inclusion criteria (Fig. 1). The inter-reviewer appraisal score was 8, indicating a high level of agreement between reviewers (Table 2). One study did not have a strong appraisal according to the criteria of the Qualitative Assessment and Review Instrument of method of quality appraisal and only the authors of one article included a positionality statement. However, the reviewers felt that the findings presented in the paper substantially added to the literature included in the review and did not exclude any studies on the basis of appraisal alone (Table 3).

**Table 2:** Inter-reviewer reliability table

Study	Number of questions in agreement	Number of questions in disagreement	Score
*Toffolon-Weiss* et al*, 2008*	5	5	5
*Schmidt-Grimminger* et al*, 2013*	8	2	8
*Bowen DJ* et al*, 2014*	8	2	8
*Clark* et al*, 2014*	10	0	10
*Henderson RJ* et al*, 2018*	9	1	9
Mean			**8**

**Table 3:** Appraisal of included studies

Citation	Q1	Q2	Q3	Q4	Q5	Q6	Q7	Q8	Q9	Q10
Bowen DJ WD. 2014.	U	Y	Y	Y	Y	N	N	Y	U	Y
Henderson RJ S-BM. 2018.	Y	Y	Y	Y	Y	N	U	Y	Y	Y
Schmidt-Grimminger D FL. 2013.	Y	Y	Y	Y	Y	N	N	Y	U	Y
Clark E. 2014.	Y	Y	Y	Y	Y	Y	Y	Y	Y	Y
Toffolon-Weiss M. 2008.	U	Y	Y	N	U	N	N	N	U	N
%	60.0	100.0	100.0	80.0	80.0	20.0	20.0	80.0	40.0	80.0

**Fig. 2:**
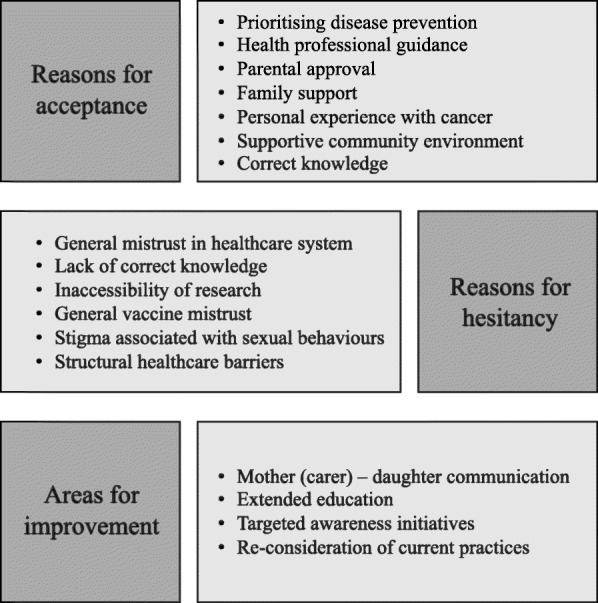
Conceptual model for HPV Vaccine acceptance and hesitancy among Indigenous populations

Studies were conducted in three countries: with Shipibo-Konibo communities in Peru; First Nations leaders, elders, and health service directors in Canada; and Alaskan Native, American Native and Northern Plains American Indian communities in the United States (Table 4). Three of the studies reported theories used in their study design, including community based participatory research [29], grounded theory [30], and trauma-informed lens [31]. Reviewers extracted 58 findings from the included articles and generated a table with each finding, illustration, and score (Supplementary file 3). The collaborative review process and synthesis of findings resulted in 17 categories, providing an appropriate base for meta-aggregation. Three overarching synthesized findings resulted from the meta-aggregation, with reviewers in agreement of all decisions (Fig. 2, Supplementary file 4).

**Table 4:** Characteristics of Included Studies

Study	Methods for data collection and analysis	Country	Phenomena of interest	Setting/context/culture	Participant characteristics and sample size	Description of main results
Bowen DJ WD. 2014.	Recruitment: flyers in public places, word of mouth, referrals from social groups Five focus groups, 90 min sessions. Recording sessions and transcribing Analysis: Data coded, analyzed and interpreted to identify emerging themes	United States of America	Attitudes and beliefs for cancer screening practices in American Indian women	American Native/ American Indian	102 participants Age range: 18–64 years Caregivers of adolescent Native American girls (for whom HPV vaccine is recommended)	Themes: 1. Disease prevention is important 2. HPV vaccine recommendations are unclear 3. Communicating with daughter 4. Confusion about HPV testing and HPV vaccination 5. Patient-provider relationship is important 6. Medical Mistrust
Henderson RJ S-BM. 2018.	Recruitment: One day event with First nations elders and leaders, presentations, discussions and sharing circles. The discussions were recorded and transcribed. Analysis: Coding of transcriptions in NVivo 10 including a thematic analysis	Canada	Barriers and facilitators for HPV vaccinations among First Nation populations	First Nations leaders, elders and health service directors	Sample Size: 24	Themes: 1. The need for a trauma informed lens 2. Role of family and community ties 3. Adapting to a changing information landscape
Schmidt-Grimminger D FL. 2013.	Community based participant research, Focus groups for qualitative data, transcription and coding of data collected Thematic analysis	United States of America	Knowledge, attitudes and beliefs related to the HPV vaccine and factors that facilitate or hinder vaccination among Alaskan Native populations	Alaskan Native groups	Sample size: 73	Themes: 1. HPV and HPV vaccine perceptions 2. Information needs and service providers 3. Barriers to HPV vaccination 4. Suggestions for improving HPV vaccination rates
Clark E. 2014.	Semi-structured interviews, thematic analysis based upon grounded theory	Peru	Knowledge, attitudes, beliefs about cervical cancer, HPV and HPV vaccine	Ucayali river basin in the Amazonian province of Ucayali; Shipibo-Konibo Indigenous Women	(*N* = 30), Women, ages 18–39, Shipibo-Konibo Indigenous,	Geographic differences in attribution of cervical cancer and importance of vaccine information for parents, although few women had heard of the HPV vaccine, all were in favour of their daughters receiving vaccination
Toffolon-Weiss M. 2008.	Focus groups, audiophiles and moderator notes on non-verbal behaviours; analysed with Atlas TI software	Alaska, USA	Parental attitudes on cervical cancer, HPV and HPV vaccine	Alaska Native parents from urban, hub and village communities	(*N* = 79), all had a 9–18 year old daughter or ward; 64 female, 15 male, age 21–61+, *N* = 28 experience in medical setting	The majority of parents were interested in having their daughters vaccinated. Accep- tance of the vaccine was primarily based on a parent’s desire to protect her/his child from cancer; while reasons for refusal revolved around trust issues and fear of unknown negative consequences of the vaccine.

### Reasons for acceptance

Many findings in this review reflect the rationale of parents or carers for why they pursued HPV vaccination for their daughter or why they wanted to. Parents identified vaccine use as a mechanism for, and result of, prioritising disease prevention [30–33]. One mother identified access to vaccines as a privilege that she did not have when she was younger [33]. Findings from two studies suggested that prioritisation of disease prevention was related to the belief that one’s daughter is susceptible to HPV infection [30, 33]: *“Maybe sometime in the future, at a young age, my daughter could become pregnant so the virus could affect her. Better said, this age [for vaccination] is good.”* [30]*.*

Personal experience with cancer was an important reason for acceptance among participants [31–33]. Many discussed a family history of cancer as a concern in terms of their child or grandchild’s health: *“For me, having a strong history of all kinds of cancers in my family, one less cancer – the vaccine could protect my daughter from at least that”* [33]. Wider support from schools, communities and families was highlighted as facilitating vaccine acceptance among participants [30, 31]. Schools were identified as safe spaces for sexual health promotion in relation to HPV vaccination because of the existing support networks between students and staff in these environments [29]. Additionally, guidance from health professionals increased vaccine acceptance among participants [30, 32], helping to provide more information or reassurance to parents or children who previously had apprehensions:

*“At first [I] was worried because ... [I] didn’t understand why [the health workers] would give her the vaccine at this age. After they explained it, [I] felt happy...that [my] daughter had received it, that she was chosen to have the vaccine.”* [30]*.*

Knowledge and a good foundational understanding of the HPV vaccine was another reason for acceptance [33]. At the completion of focus groups, researchers from one project asked participants if they would support their children getting vaccinated after having discussed it in more depth. The majority of parents agreed they would [33]. One participant shared that her daughter had done her own research on the vaccine and was eager to get it [33]. While this daughter had made the decision on her own, some parents, particularly mothers, discussed the necessity of parental approval prior to acceptance of the vaccine [32, 33]:

*“You know, it’s like, it seems like a lot of people are saying it’s their decision but in a way you know, it is up to the parent. Like you said, you can’t bring them kicking and screaming, but if I felt that, if I felt so strongly about it, which I’m not sure that I do at this point, if I felt so strongly, yeah I’d bring ‘em kicking and screaming, just like any other vaccine.”* [32]*.*

### Reasons for hesitancy

Related to vaccine acceptance, reasons for vaccine hesitancy was another synthesized finding. General mistrust in healthcare systems [30–33] and in vaccines [33] contributed to vaccine hesitancy for participants. Mistrust in healthcare systems reflected the history of maltreatment among Indigenous peoples and a lack of trusting relationships with current systems: *“Over my lifetime I’ve heard stories about Alaska Natives being used as guinea pigs and being vaccinated without their knowledge. And obviously you guys are trying to inform, but I’ve heard stories”* [33]. Indigenous health providers were hesitant to vaccinate as well, with one worrying that the vaccines were *“not natural … they are more chemicals given by the government to hurt us”* [31]. Other participants were wary of vaccine provider abilities, worrying that the vaccine may be placed incorrectly or that a trainee would be administering the vaccine [30]. One participant highlighted the importance of screening and education, providing insights into her rationale for avoiding the HPV vaccine:

*“It goes back to vaccine versus screening, that sort of thing, you know what I mean? I think that because of the way I think, on a more natural level I don’t trust drug companies, I don’t trust most drugs, or any really. Um, vaccines have side effects … this is a new vaccine, we don’t know what any long-term side effects are to it … I think I would go more for the screening and educating my child about how HPV is transmitted and not just HPV but other … sexually transmitted diseases. I think we need to teach our children, especially our daughters, how to listen to their bodies, you know, pay attention to their bodies, take responsibility for that.”* [32]*.*

Beliefs that research had not been conducted with regards to vaccine safety or efficacy, demonstrated the inaccessibility of research findings for the included communities [29, 33]. Similarly, a lack of knowledge about the HPV vaccine created hesitancy for some participants [29, 31–33]. Examples of incorrect knowledge among participants included that the vaccine could cause cancer or other diseases, that it was unavailable for men and that you had to be younger than 18 to receive it: *“If you got the shot you might get [HPV]. So I was kinda nervous … I didn’t want my niece to have a chance at getting that, so we didn’t finish it”* [29]. Several parents acknowledged that they had limited knowledge and wanted more education so that informed decisions were easier to make and they could help spread awareness to their families [29]. Indigenous health workers in one study specifically identified education for parents as the first step necessary for informed vaccination decisions [29].

Structural healthcare barriers were discussed by both participants and Indigenous health workers in terms of limited resources [29]. Limited vaccine endorsement from Indigenous health workers was highlighted by participants: *“Doctors should recommend it more, because I don’t think I ever heard about it until I was 23”* [29]. Patients additionally discussed long waiting times for appointments, healthcare provider shortages and restricted appointment lengths. Indigenous health workers identified the need for a systematic approach to result in increased uptake in communities, suggesting the possibility of working with clinic pharmacies to provide vaccine education [29].

One paper discussed the stigma associated with sexual behaviours as potential rationale for hesitancy among community members, describing the local narrative around HPV infection: *“I’ve heard my friends say, HPV is what dirty people get”* [29]. Another participant from the same study discussed how fathers could be a barrier to vaccination because they may perceive vaccination approval as endorsement for promiscuous behaviour [29].

### Areas for improvement

Through discussions around the HPV vaccine, participants from four of the included studies [29–32] identified areas that would help improve vaccine understanding, and ultimately, vaccine uptake. A commonly identified area for improvement was the need for targeted awareness initiatives for particular groups within communities, including vulnerable populations [31], community-wide programs (outside of school systems) [31] and healthcare workers [29]. As one Indigenous healthcare worker noted:

*“For us to get out there and reach these people, we have to know what we are talking about … We need to be educated on it before we can take it and present it to people in our communities.”* [29]*.*

Further, participants discussed how current initiatives are often impersonal and detached from an individual’s health: *“Doctors just throw stuff at us, so many papers [brochures]”* [31]. The importance of culturally appropriate awareness initiatives, preferably in verbal rather than written form, and ideally available in Native languages was identified as important by community members [29, 31]. Likewise, participants called for extended education practices to include whole families and communities, underscoring the importance of male voice and understanding in HPV conversations: *“I would not have any problem and would not be worried if they assured me, gave me good information and that person was trustworthy, and the information was also given to my husband”* [30].

Some participants felt that current HPV vaccination practices, specifically the suggested age for vaccination in their respective countries, should be re-considered [30–32]. Many mothers shared their beliefs that current recommendations are too young for vaccination against a sexually transmitted infection, identifying a large gap in time before their children become sexually active [32]. Within the same discussion, other mothers identified similarities between the HPV vaccine and birth control, contemplating that if a child brings up birth control it often indicates that they need it because they are having sex, at which point HPV prevention via vaccination might be too late [32]. One mother mentioned the possibility of sexual exposure at an early age, outside of one’s control, where prevention at an early age would be key [31]. The variance in beliefs and understandings of age recommendations highlights the importance of community collaboration in establishing health guidelines for each individual community. The possibility of incorporating the HPV vaccine with other infant vaccinations was also discussed:

*“It would be better if it were the same as the rest of the vaccines they give to the newborns, at three months, six months, four months. I’d prefer it more if it was like that, so that it would be more effective, just like the other vaccines. And so that there would be a way to keep track, like the other [vaccine record] cards. It would be the same and there it could integrate into that group of vaccines.”* [30]*.*

Leveraging discussions around HPV vaccination as a chance to strengthen mother or carer communication with children was discussed as important. Some participants were disappointed when they had learned that their children or grandchildren had already received the vaccine, identifying a loss of opportunity to establish and foster openness between generations around protecting one’s health [31]. Other participants felt that strong communication was often established too late to prioritise prevention, such as vaccination [32]. Many participants took the opportunity to discuss HPV vaccination, body autonomy and responsibility simultaneously with their children:

*“I left it up to the two oldest ones. I left it up to them. Sat down, got as much information material as possible in regard to the whole HPV. Went through the family history with’em, between the aunts and both sides of the family and which ones have cancer so the likelihood. You know, so, the whole DNA thing …*. *So my daughter who’s 17 years old now, she’s a smart girl, I told her this is your body and I’m not gonna to make that decision for you. Here’s the information, you know, read up, when we go to the doctor you know, for the next time, talk with them, ask as many questions as you want, and then it’s your judgment.”* [32]*.*

## Discussion

The aim of this systematic review was to explore the knowledge, beliefs, attitudes, and firsthand experiences of global Indigenous populations regarding HPV vaccinations. The findings represented in the conceptual model of reasons for vaccine acceptance and hesitancy among community members in the included studies, as well as areas for improvement, help generate insight into HPV vaccine uptake and understanding among the communities from the included studies. This review highlights the importance of community voice in design and delivery of awareness initiatives [29–31] as well as community co-creation of health recommendations for the HPV vaccine. Previous works have highlighted that vaccine-decision making is not a straightforward process with various factors impacting an individual’s decision [34]. These include perception of disease risk, vaccine risk, vaccine safety, social discourses, communication structures, knowledge, and healthcare professional recommendations [20, 21, 35, 36].

The findings align with previous explorations of Indigenous understandings and uptake of vaccinations. Intergenerational impacts of colonisation, historic maltreatment and continuing marginalisation and oppression have significantly impacted Indigenous trust in health-related services, communications, and professionals [37–40]. Synthesised findings from the included studies highlight a commonality of mistrust in healthcare systems and vaccines [30–33], with participants describing feeling like a ‘guinea pig’ when considering vaccination [33]. These feelings directly relate to historic injustices experienced by Indigenous peoples, such as medical experimentation experienced by Cree communities in Canadian residential schools [41]. Health professionals have a responsibility to educate themselves prior to providing care in communities; many non-Indigenous health workers are unaware of the oppressive history of healthcare systems and therefore do not properly understand potential vaccine hesitancy and mistrust they may encounter [38]. Interpersonal communication with practitioners is the foundation of quality care, however it is often one of the largest barriers for Indigenous peoples [40, 42]. While mistrust in healthcare is common for Indigenous communities, participants both in this qualitative review [29, 30, 32] and elsewhere [37, 43, 44] have discussed the centrality of practitioner-patient relationships and health professional guidance in promoting vaccine acceptance. Health practitioners have an ethical obligation to respectfully engage in honest conversations with Indigenous peoples about vaccines that prioritises oral forms of education [30, 31] and increases understanding for patients. For example, clinical yarning has been suggested as a mechanism to improve clinician-patient communication with Indigenous peoples in Australia that focuses on integrating cultural communication strategies with biomedical understandings of health [45]. Yarning is a traditional way of discussing important topics, with information often embedded within stories [46]. The three-pronged approach to clinical yarning includes social yarns, where clinicians find common ground and develop relationships with patients; diagnostic yarns, which aim to establish the patient’s health story through a scientific lens; and management yarns, which utilise stories as a tool to increase patient understanding and develop a collaborative management approach [45].

Related to practitioner influence on vaccine acceptance, Indigenous health workers from one study brought attention to the need for increased education [29]. Both Indigenous health workers and patients mentioned low referral rates for the HPV vaccine in this review; increasing Indigenous health worker knowledge would directly increase the frequency of vaccine recommendations and by extension, community uptake due to the influence of practitioner guidance on vaccine acceptance. Limited awareness initiatives for health workers may be related to structural barriers within healthcare systems. Similar structural barriers to those discussed [29] have been documented elsewhere as obstacles to vaccination programs [30, 39, 47–49]; specifically, limited resources and waiting times have been correlated with inaccessible vaccine programs. The synthesised finding of inaccessible research in this review aligns with the notion of perceived lack of testing identified among Métis communities discussing the H1N1 vaccine in Canada [37] and highlights the importance of making research accessible for communities with culturally relevant dissemination materials [50]. Some participants from the included studies voiced concern or disagreement with current guidelines [30–32]; co-creation of recommendations with specific communities or tailored education programming could address the misalignment of values observed [51, 52].

Mother-daughter communication was described as an area for improvement in this review [31, 32]. This reinforces how intergenerational disruptions experienced by many Indigenous communities continue to shape Indigenous health [31, 53]. Prior to colonisation, sexuality was not considered shameful for Indigenous communities in Canada; adults and elders openly discussed sexual health and taught children about their bodies. Sexuality was perceived as a gift to respect within oneself and with others [31, 53, 54]. These significant traditions provide insight to the shift in modern discourse but also provide an opportunity for awareness initiatives to strengthen communication and relationships between elders and youth. Participants from the included studies also emphasised the importance of centralising men in HPV conversations to increase understanding and family uptake of the vaccine.

Measures of vaccine coverage are challenging to determine which make it difficult to quantify the impact of population-level vaccination protection [55] and highlights the importance of qualitative research in this area. Exact HPV vaccination numbers for communities included in this review are not known, coverage levels typically report levels from the general population and hide sub-populations, including Indigenous communities [55]. Despite the limited data available for HPV vaccination coverage among Indigenous communities, data for other childhood vaccinations among Indigenous peoples have been reported as below population levels [56–58]. Researchers in both Canada and Australia are striving to improve the accuracy of vaccination coverage tracking for Indigenous communities [59, 60]. Some of the findings from this qualitative review align with previously documented barriers to HPV vaccination for both Indigenous and non-Indigenous people in Canada and the United States. For non-Indigenous people in Canada, HPV knowledge, family support, and perceived vaccine safety increase acceptance and uptake of the HPV vaccine [61]. In the United States, limited knowledge, belief that the vaccine is not effective, and uncertainty about vaccine safety have been reported as reasons for vaccine hesitancy among non-Indigenous people [62]. Multi-dose vaccine delivery and privacy challenges in school settings were not mentioned among participants from the studies included in this review but have been reported as barriers for non-Indigenous populations [63, 64].

Qualitative research enables comprehensive exploration of lived experiences, which is extremely useful for complex issues like HPV vaccination. It is important to identify and consider author subjectivity and the related impact on the creation of findings [65, 66]. However, only one article included in this review contained a reflexive stance and positionality statement. The lack of reflexivity in qualitative publications has been flagged as an area of concern to be addressed and considered by all researchers engaging in qualitative research due to the inseparable relationship between researcher subjectivity and qualitative findings [65, 66]. The findings from this review primarily focused on experiences of women and daughters, despite the recommendation for all young adults to receive the HPV vaccine [67]. Future works should explore the experiences of HPV vaccination across all genders.

### Strengths and limitations

To the best of our knowledge, this systematic review is the first to collate qualitative perspectives of HPV vaccination among Indigenous peoples at a global level. The review was completed in accordance with all relevant protocols to ensure transparency. Highlighting areas for improvement, as discussed by participants from the included studies, is a strength of this review as it provides specific areas for future programming and policy to address. In accordance with other research, this review underscored the continuing impact of colonisation for Indigenous peoples when accessing and trusting health services, with synthesised findings providing important evidence for the work needed to address the disparities resulting from oppressive policies. All but one included study had illustrations within each of the synthesised findings, underscoring the comprehensive nature of the conceptual model. Limitations include the low number of publications eligible for review within the inclusion criteria, which highlights the need for extended work in this field that prioritises Indigenous voices in health programming and service delivery, especially considering the increasing prevalence of oropharyngeal cancer as a result of HPV [68]. The included articles are only from three countries and therefore the findings from this review cannot be generalised globally across Indigenous communities. This limitation emphasises the need for more research that centralises Indigenous perspectives on HPV vaccination in other countries. Only literature published in the English language was included; a possible drawback given that some Indigenous interviews and reports may have been in Native languages.

## Conclusion

Variance in HPV vaccine uptake among Indigenous populations is well documented [20, 21, 37]. While quantitative research is essential for identifying health trends and disease spread, qualitative research is essential in exploring the stories and reasonings behind quantitative findings. Qualitative systematic reviews have the opportunity to uniquely inform policy decisions and to generate innovative solutions that successfully engage and directly benefit involved communities. Lack of knowledge is frequently correlated with lower vaccine uptake. While increased knowledge in communities would likely increase vaccine acceptance, the common sentiments expressed in this review of mistrust between individuals and healthcare systems [29, 32, 33] is deep-rooted in the colonial history of exploitation of Indigenous peoples. Educational programming will never have the capacity to resolve such profound issues. Addressing the aspects of health systems that currently function to preserve oppressive traditions is required to provide the fundamental human right of quality care to Indigenous peoples. Papers included in this review [29, 30] have highlighted various frameworks to consider when co-creating vaccine strategies with communities, such as the Ecological approach, that acknowledge the wider influences impacting vaccine-decisions and permit the development of more holistic and community-targeted initiatives.

## Supplementary Information

**Additional file 1.**

## Data Availability

All data generated or analysed during this study are included in this published article and its supplementary information files.
