# Supplementary material for: HPV vaccine: uptake and understanding among global Indigenous communities – a qualitative systematic review
Source: BMC Public Health. 2021 Nov 10;21:2062. doi: 10.1186/s12889-021-12147-z (PMC8582096; doi:10.1186/s12889-021-12147-z)
Supplement: Supplementary file 1 — Additional file 1. [file 12889_2021_12147_MOESM1_ESM.docx]

**Supplementary File 1(S1)**

Logic Grid: **Pubmed: 1286: 06/01/2021**

HPV VACCINE AND INDIGENOUS POPULATIONS

| HPV vaccine | Indigenous Populations |
| --- | --- |
| HPV[Text Word] OR "papillomaviridae"[MeSH Terms] OR “human papillomavirus”[MeSH] OR “human papillomavirus”[Text Word] OR "human papillomavirus 16"[MeSH Terms] OR human papillomavirus 16 [Text Word] OR "human papillomavirus 18" [MeSH Terms] OR human papillomavirus 18 [Text Word] OR papillomavirus[Text Word] AND vaccine* [MeSH Terms] “HPV vaccine” [Text Word] AND “qualitative” [Text Word] | ("first nation"[Text Word]) OR "first nations"[Text Word]) OR "pacific islander"[Text Word]) OR "pacific islanders"[Text Word]) OR "torres strait islander"[Text Word]) OR "torres strait islanders"[Text Word]) OR aborigin*[Text Word]) OR africa*[Text Word]) OR alaska*[Text Word]) OR aleut*[Text Word]) OR amerind*[Text Word]) OR arctic[Text Word]) OR aymara[Text Word]) OR bushmen[Text Word]) OR chukchi[Text Word]) OR chukotka*[Text Word]) OR circumpolar[Text Word]) OR eskimo*[Text Word]) OR greenland*[Text Word]) OR hmong[Text Word]) OR indian*[Text Word]) OR indigen*[Text Word]) OR inuit*[Text Word]) OR inupiaq[Text Word]) OR inupiat[Text Word]) OR khanty[Text Word]) OR maori*[Text Word]) OR mapuche[Text Word]) OR metis[Text Word]) OR native*[Text Word]) OR navaho*[Text Word]) OR navajo*[Text Word]) OR nenets[Text Word]) OR quechua[Text Word]) OR saami[Text Word]) OR sami[Text Word]) OR samoan*[Text Word]) OR siberia*[Text Word]) OR skolt[Text Word]) OR tribal[Text Word]) OR tribe*[Text Word]) OR xingu*[Text Word]) OR yup’ik[Text Word]) OR yupik[Text Word]) OR zuni[Text Word]) OR "African continental ancestry group"[Mesh]) OR "African continental ancestry group"[Mesh]) OR "Asian continental ancestry group"[Mesh]) OR "Health Services, Indigenous"[Mesh]) OR "Oceanic ancestry group"[Mesh]) OR "arctic regions"[Mesh]) OR "ethnic groups"[mesh] |

Logic Grid: **EMBASE: 182: 06/01/2021**

| HPV vaccine | Indigenous Populations |
| --- | --- |
| ('human papillomavirus type 16':ti,ab,kw OR 'human papillomavirus type 18':ti,ab,kw OR 'wart virus':ti,ab,kw OR 'oral human papillomavirus infection':ti,ab,kw OR 'hpv':ti,ab,kw AND ‘HPV vaccine’:ti,ab,kw AND ‘qualitative’:ti,ab,kw | 'indigenous population':ti,ab,kw OR 'indigenous people':ti,ab,kw OR 'Samoan (people)':ti,ab,kw OR 'First Nation':ti,ab,kw OR 'Pacific Islander':ti,ab,kw OR 'Torres Strait Islander':ti,ab,kw OR 'Black person':ti,ab,kw OR 'Alaska Native':ti,ab,kw OR 'Aleut (people)':ti,ab,kw OR 'Amerind people':ti,ab,kw OR 'Aymara (people)':ti,ab,kw OR 'Chukchi (people)':ti,ab,kw OR 'Chukotka':ti,ab,kw OR 'Eskimo-Aleut people':ti,kw,ab OR 'Greenland':ti,ab,kw OR 'Hmong (people)':ti,kw,ab OR 'Indian':ti,ab,kw OR 'indigenous people':ti,kw,ab OR 'Inuit':ti,kw,ab OR 'Inupiat (people)':ti,kw,ab OR 'Khanty (people)':ti,ab,kw OR 'Maori (people)':ti,kw,ab OR 'Mapuche (people)':ti,kw,ab OR 'indigenous people':ti,kw,ab OR 'Navajo (people)':ti,kw,ab OR 'Nenets (people)':ti,kw,ab OR 'Quechua (people)':ti,kw,ab OR 'Sami (people)':ti,kw,ab OR 'Yupik (people)':ti,ab,kw OR 'Zuni (people)':ti,ab,kw OR 'Asian continental ancestry group':ti,ab,kw OR 'Oceanic ancestry group':ti,ab,kw OR 'Arctic':ti,ab,kw OR 'ethnic group':ti,kw,ab |

Logic Grid **SCOPUS: 402: 06/01/2021**

| HPV Vaccine | Indigenous Populations |
| --- | --- |
| TITLE-ABS-KEY (HPV) OR TITLE-ABS-KEY (papillomaviridae) OR TITLE-ABS-KEY (human papillomavirus) OR TITLE-ABS-KEY (human papillomavirus type 16) OR TITLE-ABS-KEY (human papillomavirus type 18) AND TITLE-ABS-KEY (HPV vaccine) AND TITLE-ABS-KEY (qualitative) | TITLE-ABS-KEY ( indigenous  AND population )  OR  TITLE-ABS-KEY ( ethnic  AND groups )  OR  TITLE-ABS-KEY ( aborigin )  OR  TITLE-ABS-KEY ( torres  AND strait  AND islander )  OR  TITLE-ABS-KEY ( black  AND person ) |

**Web of Science: 964: 06/01/2021**

| HPV vaccine | Indigenous Populations |
| --- | --- |
| (HPV OR human papillomavirus OR human papillomavirus type 16 OR human papillomavirus type 18 OR Oral HPV OR wart virus AND HPV vaccine AND qualitative) | **TOPIC:** ((first nation OR first nations OR pacific islander OR pacific islanders OR torres strait islander OR torres strait islanders OR aborigin* OR africa* OR alaska* OR aleut* OR amerind* OR arctic OR aymara OR bushmen OR chukchi OR chukotka* OR circumpolar OR eskimo* OR greenland* OR hmong OR indian* OR indigen* OR inuit* OR inupiaq OR inupiat OR khanty OR maori* OR mapuche OR metis OR native* OR navaho* OR navajo* OR nenets OR quechua OR saami OR sami OR samoan* OR siberia* OR skolt OR tribal OR tribe* OR xingu* OR yup’ik OR yupik OR zuni OR African continental ancestry group OR African continental ancestry group OR Asian continental ancestry group OR Health Services, Indigenous OR Oceanic ancestry group OR arctic regions OR ethnic groups)) |

**Supplementary File 2 (S2)**

**JBI SUMARI appraisal tool questionnaire for qualitative systematic reviews**

1. Is there congruity between the stated philosophical perspective and research methodology?
2. Is there congruity between the research methodology and the research question or objectives?
3. Is there congruity between the research methodology and the methods used to collect the data?
4. Is there congruity between the research methodology and the representation and analysis of data?
5. Is there congruity between the research methodology and the interpretation of results?
6. Is there a statement placing the researcher culturally or theoretically?
7. Is the influence of the research on the researcher and vice versa, addressed?
8. Are the participants and their voices adequately represented?
9. Is there research ethical according to current criteria or, for recent studies, and there evidence for ethical approval by an appropriate body?
10. Do the conclusions drawn in the research report flow from the analysis, or interpretation of the data?

**Supplementary file (S3)**

**Study findings, corresponding illustration, and score (unequivocal, credible or not credible)**

| **Study: Clark 2014** | |
| --- | --- |
| Finding | Perceptions of the HPV Vaccine (U) |
| Illustration | "At first [I] was worried because ... [I] didn’t understand why they would give her the vaccine at this age. After they explained it, [I] felt happy...that [my] daughter had received it, that she was chosen to have the vaccine." |
| Finding | Family perceptions of the HPV Vaccine (C) |
| Illustration | When asked, all but one participant said they felt their families would be supportive of the vaccine as well. One woman said it didn’t matter to her if her family agreed or disagreed, that the decision to vaccinate was between her and her daughter. |
| Finding | Importance of Informing Parents, including fathers (U) |
| Illustration | "I would not have any problem and would not be worried if they assured me, gave me good information and that person was trustworthy, and the information was also given to my husband." |
| Finding | Ideal age for HPV Vaccination - agree with current guidelines (U) |
| Illustration | "Maybe sometime in the near future, at a young age, my daughter could become pregnant. So the virus could affect her. Better said, this age [for vaccination] is good." |
| Finding | Ideal age for HPV Vaccination - disagree with current guidelines (U) |
| Illustration | "It would be better if it were the same as the rest of the vaccines they give to the newborns, at three months, six months, four months. I’d prefer it more if it was like that, so that it would be more effective, just like the other vaccines. And so that there would be a way to keep track, like the other [vaccine record] cards. It would be the same and there it could integrate into that group of vaccines." |
| Finding | Desire to ensure vaccinations done correctly (U) |
| Illustration | "Well, they could place the vaccine badly, or they could make an error about the medicine, or the person who places it could be a trainee." |
| Finding | Prioritisation of daughter health (U) |
| Illustration | "Yes, I too said that with [my daughter] when she was little. “Ay, I don’t want them to vaccinate her because she will cry.” But later I though, “I’m wrong.” Look, it’s okay that she cries. Crying is not going to kill her. It would be worse if a disease got her." |
| **Study: Bowen DJ 2014** | |
| Finding | Disease Prevention is Important (C) |
| Illustration | Only a few of the women consistently engaged in preventive behaviors or commented that prevention was not important or preferable to treatment |
| Finding | HPV Vaccine Recommendations are Unclear (C) |
| Illustration | Many women expressed the idea that 9 years old was too young to get a vaccine that protected against a sexually transmitted disease because the child was still a few years away from having sex, and it was at that later point that the decision should be made about vaccination or not. |
| Finding | HPV Vaccine Recommendations are Unclear (C) |
| Illustration | Some women pointed out that by the older age, it might be too late, and that if girls had to bring up a vaccine when they were considering having sex, it would operate the same as birth control in that if a child brings up birth control, it means that she is having sex and needs it. |
| Finding | Communicating with Daughter Varied (C) |
| Illustration | Native women indicated that by the time that good mother–daughter communication about sexual issues occurred, it would be too late, as exposure would have already occurred. |
| Finding | Communicating with Daughter Varied (U) |
| Illustration | "I left it up to the two oldest ones. I left it up to them. Sat down, got as much information material as possible in regards to the whole HPV. Went through the family history with’em, between the aunts and both sides of the family and which ones have cancer so the likelihood. You know, so, the whole DNA thing…. So my daughter who’s 17 years old now, she’s a smart girl, I told her “this is your body and I’m not gonna to make that decision for you. Here’s the information, you know, read up, when we go to the doctor you know, for the next time, talk with them, ask as many questions as you want, and then it’s your judgment" |
| Finding | Communicating with Daughter Varied (U) |
| Illustration | "When we sat down with xxxx (daughter), and I went through thing well why are they getting the shot,… I explained to her about the virus. Well how do you get it? I said ‘well it’s sexually transmitted’ now she picked up – on – that and wanted to discuss it with me, and then I carry on. I think she, at 9 y/o, she knows about sex… and so I explained to her about that. And, you know, I said you know ‘you’re 9 years old’ you know. I said, you know I’m not gonna do it now, but when you get the shot, get the shot. She said ‘No, I’ll wait!’ But I explained everything to her and you know, given her pediatrician explain it to her. |
| Finding | Communicating with Daughter Varied (U) |
| Illustration | "It seems to me that this vaccine, like when you see the commercials and all it’s like “talk to your child”. Well we don’t have the opportunity to talk to our children about other things that we’ve had to give them. You know, it’s like, it seems like a lot of people are saying it’s their decision but in a way you know, it is up to the parent. Like you said, you can’t bring them kicking and screaming, but if I felt that, if I felt so strongly about it, which I’m not sure that I do at this point, if I felt so strongly, yeah I’d bring ‘em kicking and screaming, just like any other vaccine." |
| Finding | Confusion about HPV Testing and HPV Vaccination (U) |
| Illustration | "It’s, I mean, there are percentages, there’s risk and everything, but that adult person, as to whether you want to take that risk, or you don’t… or you run a risk of em getting the disease. That’s what it comes down to me. It’s the risk of the drug against the risk of the disease itself." |
| Finding | Confusion about HPV testing and HPV Vaccination (N) |
| Illustration | "You know, …when they come to me, you know, I just, I don’t like putting any foreign objects, substance in my body, that’s man made…I don’t know, I just can’t, I can’t do it. When it comes to the innovation shots with the kids, you know, some of them I think it’s, like small pox. come on, people, …why are you shooting my kid up with this live virus, what’s the matter with you? Then the whole circumstances, you know, …well gee, guess what? you have the innovation right there, so why put my kid, even though the risk factor’s so low but, you know, they could be the 1 in you know 100,000 deaths or could come down with, now with some of the shots they are saying that it has to do with autism later in life. Participant—The mercury? I had a very hard time with that also. Especially with my son because it’s, the rates are higher in boys. I had a really, I held off for longer than you’re supposed to, because I just didn’t feel comfortable." |
| Finding | Confusion about HPV testing and HPV Vaccination (U) |
| Illustration | "You know, …when they come to me, you know, I just, I don’t like putting any foreign objects, substance in my body, that’s man made…I don’t know, I just can’t, I can’t do it. When it comes to the innovation shots with the kids, you know, some of them I think it’s, like small pox. come on, people, …why are you shooting my kid up with this live virus, what’s the matter with you? Then the whole circumstances, you know, …well gee, guess what? you have the innovation right there, so why put my kid, even though the risk factor’s so low but, you know, they could be the 1 in you know 100,000 deaths or could come down with, now with some of the shots they are saying that it has to do with autism later in life. Participant—The mercury? I had a very hard time with that also. Especially with my son because it’s, the rates are higher in boys. I had a really, I held off for longer than you’re supposed to, because I just didn’t feel comfortable." |
| Finding | Medical Mistrust (U) |
| Illustration | "It’s a little bit off what you’re just asking us. It goes back to vaccine versus screening, that sort of thing, you know what I mean? I think that because of the way I think, on a more natural level I don’t trust drug companies, I don’t trust most drugs, or any really. Um, vaccines have side effects and could cause, this is a new vaccine, we don’t know what any long-term side effects are to it. So I think in that way, if I had a daughter that was that young now. I have, my daughter’s 33, 34. But SHE has a daughter. I think I would go more for the screening and educating my child about how HPV is transmitted and not just HPV but other …sexually transmitted diseases. I think we need to teach our children, especially our daughters, how to listen to their bodies, you know, pay attention to their bodies, take responsibility for that" |
| **Study: Toffolon-Weiss 2008** | |
| Finding | Daughter decision making (U) |
| Illustration | “My daughter is 17, and she’s the one who went out, did her research on the shot, and she’s been patiently waiting for it.” |
| Finding | Maternal decision making (C) |
| Illustration | The majority of mothers in the Alaskan focus groups said that they alone made the decision to vaccinate their children against a disease. Some said that they made the decision in conjunction with their spouses and a few said that they involved their daughters and spouses in the decision-making. |
| Finding | Acceptance of vaccine (C) |
| Illustration | When asked in a “round-robin” fashion at the end of the focus group whether they would get their children vaccinated, the majority of parents answered affirmatively. |
| Finding | Health and safety concerns (U) |
| Illustration | “I see it as just part of being a mom and wanting to protect your child against cancer.” |
| Finding | Personal experience with cancer/HPV (U) |
| Illustration | “For me, having a strong history of all kinds of cancers in my family, one less cancer – the vaccine could protect my daughter from at least that.” |
| Finding | Belief in usefulness of vaccines (U) |
| Illustration | “I didn’t have all that privilege of getting all of those kinds of vaccines. Now that they are coming up with good kinds of things I would give my kids the privilege to get them.” |
| Finding | In case of sexual assault exposure child will be protected (C) |
| Illustration | Another theme that was mentioned by at least one parent in each community was that often sexual exposure was not under the control of the young woman, as in the case of rape, and this vaccine would offer the young woman protection from HPV. |
| Finding | Not encouraging promiscuity (U) |
| Illustration | “I don’t think it’ll encourage my daughter to go out and have sex. I don’t want her to have sex now. She’s 14. I hope she has sex in the future and has kids and lives a normal life, but I don’t think it will encourage her to go act irrationally.” |
| Finding | Belief that child is susceptible to HPV (C) |
| Illustration | Parents appeared to view the vaccine strictly from a health-related perspective and to accept that their daughters would eventually be sexually active as they grew older and would become susceptible to HPV |
| Finding | Not enough research has been done (U) |
| Illustration | “I don’t like to be the first to use a new vaccine. That makes me uncomfortable that it hasn’t been used by a lot of people yet. Some side effects may turn up that they don’t know about until they vaccinate a whole bunch of kids.” |
| Finding | Need more information (U) |
| Illustration | “I lean towards it, it sounds like a good thing. I just wish there was a lot more information, and I wish there was a lot more information from somebody other than Merck.” |
| Finding | Mistrust about vaccines (U) |
| Illustration | “Well, just like she was saying, there’s that risk of introducing something into your body that you probably wouldn’ t have contracted, but then you introduce it and you get it. That’s my fear.” |
| Finding | Don't want to be used as a "guinea pig" (U) |
| Illustration | “Over my lifetime I’ve heard stories about Alaska Natives being used as guinea pigs and being vaccinated without their knowledge. And obviously you guys are trying to inform, but I’ve heard stories.” |
| **Study: Henderson RJ 2018** | |
| Finding | Cancer and HPV experiences (C) |
| Illustration | Recognizing a complex sequence of risks that may heighten vulnerability, the participant ensured that her daughter received the HPV vaccine at school, wishing only that it had been available sooner. |
| Finding | Family and Community Support (U) |
| Illustration | "I am learning a lot in these workshops. My mother died of stomach cancer, my sister of stomach cancer. I had 5 girls, and 4 of them went through breast cancer. My oldest daughter, her cancer spread. When they were younger I made sure they all got their needles. But, you know, I have never had a workshop like this. If I get a cold, I can fight it off. When I got those needles, I was told I was able to fight the sicknesses; it won't kill you—that is what I was told. This is really good for my grandchildren; I will take this message home to my family. I have two nurses in my family, they probably know about it, but this is a really good thing I am still learning…[*speaking in Cree] *I was worried the white people would not take care of us, but they have so far [group laughs]. We need to talk to young ladies about how to take care of themselves" |
| Finding | Trauma Informed Lens (C) |
| Illustration | Those in the child welfare system were described as having few supports to learn anything about their own bodies, let alone to have anyone following up whether they receive the HPV vaccine within school-based programs. |
| Finding | Family and Community (U) |
| Illustration | "The health centre sent out a notification and a consent form, and they listed the benefits and risks…and I paid more attention to the risks, and I decided not to allow her to be vaccinated, because as a parent I needed to do what was best for my children” |
| Finding | Family and Community (U) |
| Illustration | “There are anti-bullying programs…and there are cultural programs; some kids are brave and some are afraid of getting immunized, but all the children support each other” |
| Finding | Family and Community (C) |
| Illustration | This supportive environment among youth receiving the vaccine was encouraged in some communities by celebrating the event of vaccination itself, with a meal and acknowledging the support that youth provided to one another |
| Finding | Family and Community (C) |
| Illustration | Some speakers were disappointed to learn after the fact that their grandchild had been vaccinated, sensing that they had lost an opportunity to discuss with the youth issues related to sexual intercourse, to foster the kind of openness between generations believed to be protective of health. These participants believed that health providers or teachers could provide some education |
| Finding | Family and Community (C) |
| Illustration | Others could appreciate that it might be difficult from a school's perspective to educate in detail all families about HPV, but these speakers proposed that school-based programs alone were not entirely effective, and instead require outreach to older generations as well, “to get the word out” |
| Finding | Family and Community (C) |
| Illustration | The importance of verbal forms of education was among the strongest themes, underscoring that prevention without relationships would be unlikely to improve HPV vaccine uptake or related health outcomes. |
| Finding | Changing Information Landscapes (C) |
| Illustration | One health director who was herself FN had not approved the HPV vaccine for her own daughter, believing at the time that vaccines are perhaps “not natural, that they are more chemicals given by the government to hurt us”. This perspective highlights mistrust in health systems even among Indigenous providers, some who may feel that health promotion is often impersonal and uninvested in one's actual health, where “doctors just throw stuff at us, so many papers [brochures]” |
| **Study: Schmidt-Grimminger D 2013** | |
| Finding | Information Needs and service Provision (U) |
| Illustration | “I still think people still have a lot of questions, they are really unsure. A question I had was how long did they do research on that vaccine to determine the safety.” |
| Finding | Information Needs and Service Provision (U) |
| Illustration | "For us to get out there and reach these people, we have to know what we are talking about…We need to be educated on it before we can take it and present it to people in our communities….” |
| Finding | Information Needs and Service Provision (C) |
| Illustration | Several parents noted that they did not feel knowledgeable, but wanted information so they could make informed decisions in order to educate their daughters and nieces about HPV. |
| Finding | HPV and HPV awareness and perceptions (C) |
| Illustration | Several parents revealed a perception that the vaccine was thought to potentially cause cancer or disease and that HPV and cervical cancer were hereditary and had a genetic cause. |
| Finding | HPV and HPV Awareness and perceptions (U) |
| Illustration | “What I've heard is the stuff on the commercials, you know. Get your shot…oh, you're 18…I missed that shot. I'm not 18 no more.” |
| Finding | HPV and HPV awareness and Perceptions (C) |
| Illustration | Within the young adult and tribal healthcare providers, there was confusion about whether or not HPV was something men could get. |
| Finding | Barriers (U) |
| Illustration | “I also learned about autism. And I started to read about vaccinations…was getting scary about getting these vaccinations.” |
| Finding | Barriers (U) |
| Illustration | “if you got the shot you might get it. So I was kinda nervous…I didn't want my niece to have a chance at getting that, so we didn't finish it.” |
| Finding | Barriers (U) |
| Illustration | However, participants did note there were negative perceptions about HPV in the community that could be a potential barrier. One participant noted, “I've heard my friends say, HPV is what dirty people get.” |
| Finding | Barriers (U) |
| Illustration | Tribal healthcare providers discussed the parent barrier as a need to provide more education, “it's got to be the number one thing, is letting them know what it is and how they can get the help and everything about it. The education is that first step.” |
| Finding | Barriers (U) |
| Illustration | “the father will be the barrier, because he thinks it gives them the go ahead to be promiscuous.” |
| Finding | Barriers (C) |
| Illustration | resource issues within the clinic were also identified in both the parent and IHS focus groups. Parents noted providers not recommending the vaccine or lengthy waiting times to get an appointment. IHS providers also recognized resource constraints within the clinic. For example, several participants noted concerns with the amount of time they had with patients and shortages of providers. |
| Finding | Improving Vaccination Rates (U) |
| Illustration | Young adults also recommended education in the school and recommended beginning in junior high or younger, and made suggestions such as, “have some nurses come in [and] do different classes.” |
| Finding | Improving Vaccination Rates (U) |
| Illustration | Parents also suggested education during health classes but also noted the possibility of vaccinating at the school, “if they get the shot in school, the first month that school started, by the time school was out those kids would be completely through.” |
| Finding | Improving Vaccination Rates (C) |
| Illustration | The groups mentioned education through venues such as health fairs, radio announcements, and posters. The tribal and IHS healthcare providers discussed the importance of culturally appropriate education and outreach. For example, the tribal healthcare providers noted that some materials should be in their native language (Lakota) in order to successfully conduct outreach to elders of the community who are important opinion leaders in their community. Similarly, the IHS providers noted that they could provide more educational materials in the clinic but stated that it would be better if they were culturally specific to the Northern Plain |
| Finding | Improving Vaccination Rates (C) |
| Illustration | “doctors should recommend it more, cause I don't think I ever heard about it until I was 23.” The IHS healthcare providers said that a more systematic approach was needed to increase the uptake of the vaccine. For example, they noted the possibility of working with the clinic pharmacy to provide counselling and the vaccine. |

**Supplemental File 4: Illustrations from all the included studies arranged according to synthesized findings and categories under the conceptual model**

| Categories | Synthesized Finding | Illustration |
| --- | --- | --- |
| Reasons for acceptance | Prioritising disease prevention | "Maybe sometime in the near future, at a young age, my daughter could become pregnant. So the virus could affect her. Better said, this age [for vaccination] is good." (Clark, 2014) |
|  |  | "Yes, I too said that with [my daughter] when she was little. “Ay, I don’t want them to vaccinate her because she will cry.” But later I thought, “I’m wrong.” Look, it’s okay that she cries. Crying is not going to kill her. It would be worse if a disease got her." (Clark, 2014) |
|  |  | Only a few of the women consistently engaged in preventive behaviors or commented that prevention was not important or preferable to treatment. (Bowen, 2014) |
|  |  | “I see it as just part of being a mom and wanting to protect your child against cancer.” (Toffolon-Weiss, 2008) |
|  |  | “For me, having a strong history of all kinds of cancers in my family, one less cancer – the vaccine could protect my daughter from at least that.” (Toffolon-Weiss, 2008) |
|  |  | “I didn’t have all that privilege of getting all of those kinds of vaccines. Now that they are coming up with good kinds of things I would give my kids the privilege to get them.” (Toffolon-Weiss, 2008) |
|  |  | Another theme that was mentioned by at least one parent in each community was that often sexual exposure was not under the control of the young woman, as in the case of rape, and this vaccine would offer the young woman protection from HPV. (Toffolon-Weiss, 2008) |
|  |  | “I don’t think it’ll encourage my daughter to go out and have sex. I don’t want her to have sex now. She’s 14. I hope she has sex in the future and has kids and lives a normal life, but I don’t think it will encourage her to go act irrationally.” (Toffolon-Weiss, 2008) |
|  |  | Parents appeared to view the vaccine strictly from a health-related perspective and to accept that their daughters would eventually be sexually active as they grew older and would become susceptible to HPV. (Toffolon-Weiss, 2008) |
|  |  | Recognizing a complex sequence of risks that may heighten vulnerability, the participant ensured that her daughter received the HPV vaccine at school, wishing only that it had been available sooner. (Henderson, 2018) |
|  | Health professional guidance | "At first [I] was worried because ... [I] didn’t understand why they would give her the vaccine at this age. After they explained it, [I] felt happy...that [my] daughter had received it, that she was chosen to have the vaccine." (Clark, 2014) |
|  |  | "I would not have any problem and would not be worried if they assured me, gave me good information and that person was trustworthy, and the information was also given to my husband." (Clark, 2014) |
|  |  | "When we sat down with xxxx (daughter), and I went through thing well why are they getting the shot,… I explained to her about the virus. Well how do you get it? I said ‘well it’s sexually transmitted’ now she picked up – on – that and wanted to discuss it with me, and then I carry on. I think she, at 9 y/o, she knows about sex… and so I explained to her about that. And, you know, I said you know ‘you’re 9 years old’ you know. I said, you know I’m not gonna do it now, but when you get the shot, get the shot. She said ‘No, I’ll wait!’ But I explained everything to her and you know, given her pediatrician explain it to her.” (Bowen, 2014) |
|  | Parental approval | "It seems to me that this vaccine, like when you see the commercials and all it’s like “talk to your child”. Well we don’t have the opportunity to talk to our children about other things that we’ve had to give them. You know, it’s like, it seems like a lot of people are saying it’s their decision but in a way you know, it is up to the parent. Like you said, you can’t bring them kicking and screaming, but if I felt that, if I felt so strongly about it, which I’m not sure that I do at this point, if I felt so strongly, yeah I’d bring ‘em kicking and screaming, just like any other vaccine." (Bowen, 2014) |
|  |  | The majority of mothers in the Alaskan focus groups said that they alone made the decision to vaccinate their children against a disease. Some said that they made the decision in conjunction with their spouses and a few said that they involved their daughters and spouses in the decision-making. (Toffolon-Weiss, 2008) |
|  | Family support | When asked, all but one participant said they felt their families would be supportive of the vaccine as well. One woman said it didn’t matter to her if her family agreed or disagreed, that the decision to vaccinate was between her and her daughter. (Clark, 2014) |
|  | Personal experience with cancer | “For me, having a strong history of all kinds of cancers in my family, one less cancer – the vaccine could protect my daughter from at least that.” (Toffolon-Weiss, 2008) |
|  |  | "I am learning a lot in these workshops. My mother died of stomach cancer, my sister of stomach cancer. I had 5 girls, and 4 of them went through breast cancer. My oldest daughter, her cancer spread. When they were younger I made sure they all got their needles. But, you know, I have never had a workshop like this. If I get a cold, I can fight it off. When I got those needles, I was told I was able to fight the sicknesses; it won't kill you—that is what I was told. This is really good for my grandchildren; I will take this message home to my family. I have two nurses in my family, they probably know about it, but this is a really good thing I am still learning…[*speaking in Cree] *I was worried the white people would not take care of us, but they have so far [group laughs]. We need to talk to young ladies about how to take care of themselves." (Henderson, 2018) |
|  | Supportive community environment | “There are anti-bullying programs…and there are cultural programs; some kids are brave and some are afraid of getting immunized, but all the children support each other.” (Henderson, 2018) |
|  |  | This supportive environment among youth receiving the vaccine was encouraged in some communities by celebrating the event of vaccination itself, with a meal and acknowledging the support that youth provided to one another. (Henderson, 2018) |
|  |  | Young adults also recommended education in the school and recommended beginning in junior high or younger, and made suggestions such as, “have some nurses come in [and] do different classes.” (Schmidt-Grimminger, 2013) |
|  |  | Parents also suggested education during health classes but also noted the possibility of vaccinating at the school, “if they get the shot in school, the first month that school started, by the time school was out those kids would be completely through.” (Schmidt-Grimminger, 2013) |
|  | Correct knowledge | “My daughter is 17, and she’s the one who went out, did her research on the shot, and she’s been patiently waiting for it.” (Toffolon-Weiss, 2008) |
|  |  | When asked in a “round-robin” fashion at the end of the focus group whether they would get their children vaccinated, the majority of parents answered affirmatively. (Toffolon-Weiss, 2008) |
| Reasons for hesitancy | General mistrust in healthcare system | "Well, they could place the vaccine badly, or they could make an error about the medicine, or the person who places it could be a trainee." (Clark, 2014) |
|  |  | "You know, …when they come to me, you know, I just, I don’t like putting any foreign objects, substance in my body, that’s man made…I don’t know, I just can’t, I can’t do it. When it comes to the innovation shots with the kids, you know, some of them I think it’s, like small pox. come on, people, …why are you shooting my kid up with this live virus, what’s the matter with you? Then the whole circumstances, you know, …well gee, guess what? you have the innovation right there, so why put my kid, even though the risk factor’s so low but, you know, they could be the 1 in you know 100,000 deaths or could come down with, now with some of the shots they are saying that it has to do with autism later in life. Participant—The mercury? I had a very hard time with that also. Especially with my son because it’s, the rates are higher in boys. I had a really, I held off for longer than you’re supposed to, because I just didn’t feel comfortable." (Bowen, 2014) |
|  |  | "It’s a little bit off what you’re just asking us. It goes back to vaccine versus screening, that sort of thing, you know what I mean? I think that because of the way I think, on a more natural level I don’t trust drug companies, I don’t trust most drugs, or any really. Um, vaccines have side effects and could cause, this is a new vaccine, we don’t know what any long-term side effects are to it. So I think in that way, if I had a daughter that was that young now. I have, my daughter’s 33, 34. But SHE has a daughter. I think I would go more for the screening and educating my child about how HPV is transmitted and not just HPV but other …sexually transmitted diseases. I think we need to teach our children, especially our daughters, how to listen to their bodies, you know, pay attention to their bodies, take responsibility for that.” (Bowen, 2014) |
|  |  | “Over my lifetime I’ve heard stories about Alaska Natives being used as guinea pigs and being vaccinated without their knowledge. And obviously you guys are trying to inform, but I’ve heard stories.” (Toffolon-Weiss, 2008) |
|  |  | One health director who was herself FN had not approved the HPV vaccine for her own daughter, believing at the time that vaccines are perhaps “not natural, that they are more chemicals given by the government to hurt us”. This perspective highlights mistrust in health systems even among Indigenous providers, some who may feel that health promotion is often impersonal and uninvested in one's actual health, where “doctors just throw stuff at us, so many papers [brochures].” (Henderson, 2018) |
|  | Lack of correct knowledge | "It’s, I mean, there are percentages, there’s risk and everything, but that adult person, as to whether you want to take that risk, or you don’t… or you run a risk of em getting the disease. That’s what it comes down to me. It’s the risk of the drug against the risk of the disease itself." (Bowen, 2014) |
|  |  | “I lean towards it, it sounds like a good thing. I just wish there was a lot more information, and I wish there was a lot more information from somebody other than Merck.” (Toffolon-Weiss, 2008) |
|  |  | "The health centre sent out a notification and a consent form, and they listed the benefits and risks…and I paid more attention to the risks, and I decided not to allow her to be vaccinated, because as a parent I needed to do what was best for my children.” (Henderson, 2018) |
|  |  | Several parents noted that they did not feel knowledgeable, but wanted information so they could make informed decisions in order to educate their daughters and nieces about HPV. (Schmidt-Grimminger, 2013) |
|  |  | Several parents revealed a perception that the vaccine was thought to potentially cause cancer or disease and that HPV and cervical cancer were hereditary and had a genetic cause. (Schmidt-Grimminger, 2013) |
|  |  | “What I've heard is the stuff on the commercials, you know. Get your shot…oh, you're 18…I missed that shot. I'm not 18 no more.” (Schmidt-Grimminger, 2013) |
|  |  | Within the young adult and tribal healthcare providers, there was confusion about whether or not HPV was something men could get. (Schmidt-Grimminger, 2013) |
|  |  | “if you got the shot you might get it. So I was kinda nervous…I didn't want my niece to have a chance at getting that, so we didn't finish it.” (Schmidt-Grimminger, 2013) |
|  |  | Tribal healthcare providers discussed the parent barrier as a need to provide more education, “it's got to be the number one thing, is letting them know what it is and how they can get the help and everything about it. The education is that first step.” (Schmidt-Grimminger, 2013) |
|  | Inaccessibility of research | “I don’t like to be the first to use a new vaccine. That makes me uncomfortable that it hasn’t been used by a lot of people yet. Some side effects may turn up that they don’t know about until they vaccinate a whole bunch of kids.” (Toffolon-Weiss, 2008) |
|  |  | “I still think people still have a lot of questions, they are really unsure. A question I had was how long did they do research on that vaccine to determine the safety.” (Schmidt-Grimminger, 2013) |
|  | General vaccine mistrust | “Well, just like she was saying, there’s that risk of introducing something into your body that you probably wouldn’ t have contracted, but then you introduce it and you get it. That’s my fear.” (Toffolon-Weiss, 2008) |
|  |  | “Over my lifetime I’ve heard stories about Alaska Natives being used as guinea pigs and being vaccinated without their knowledge. And obviously you guys are trying to inform, but I’ve heard stories.” (Toffolon-Weiss, 2008) |
|  |  | “I also learned about autism. And I started to read about vaccinations…was getting scary about getting these vaccinations.” (Schmidt-Grimminger, 2013) |
|  | Stigma associated with sexual behaviours | However, participants did note there were negative perceptions about HPV in the community that could be a potential barrier. One participant noted, “I've heard my friends say, HPV is what dirty people get.” (Schmidt-Grimminger, 2013) |
|  |  | “The father will be the barrier, because he thinks it gives them the go ahead to be promiscuous.” (Schmidt-Grimminger, 2013) |
|  | Structural healthcare barriers | Resource issues within the clinic were also identified in both the parent and IHS focus groups. Parents noted providers not recommending the vaccine or lengthy waiting times to get an appointment. IHS providers also recognized resource constraints within the clinic. For example, several participants noted concerns with the amount of time they had with patients and shortages of providers. (Schmidt-Grimminger, 2013) |
|  |  | “Doctors should recommend it more, cause I don't think I ever heard about it until I was 23.” The IHS healthcare providers said that a more systematic approach was needed to increase the uptake of the vaccine. For example, they noted the possibility of working with the clinic pharmacy to provide counselling and the vaccine. (Schmidt-Grimminger, 2013) |
| Areas for improvement | Mother (carer) – daughter communication | Native women indicated that by the time that good mother–daughter communication about sexual issues occurred, it would be too late, as exposure would have already occurred. (Bowen, 2014) |
|  |  | "I left it up to the two oldest ones. I left it up to them. Sat down, got as much information material as possible in regards to the whole HPV. Went through the family history with’em, between the aunts and both sides of the family and which ones have cancer so the likelihood. You know, so, the whole DNA thing…. So my daughter who’s 17 years old now, she’s a smart girl, I told her “this is your body and I’m not gonna to make that decision for you. Here’s the information, you know, read up, when we go to the doctor you know, for the next time, talk with them, ask as many questions as you want, and then it’s your judgment." (Bowen, 2014) |
|  |  | "When we sat down with xxxx (daughter), and I went through thing well why are they getting the shot,… I explained to her about the virus. Well how do you get it? I said ‘well it’s sexually transmitted’ now she picked up – on – that and wanted to discuss it with me, and then I carry on. I think she, at 9 y/o, she knows about sex… and so I explained to her about that. And, you know, I said you know ‘you’re 9 years old’ you know. I said, you know I’m not gonna do it now, but when you get the shot, get the shot. She said ‘No, I’ll wait!’ But I explained everything to her and you know, given her pediatrician explain it to her.” (Bowen, 2014) |
|  |  | Some speakers were disappointed to learn after the fact that their grandchild had been vaccinated, sensing that they had lost an opportunity to discuss with the youth issues related to sexual intercourse, to foster the kind of openness between generations believed to be protective of health. These participants believed that health providers or teachers could provide some education. (Henderson, 2018) |
|  | Extended education | "I would not have any problem and would not be worried if they assured me, gave me good information and that person was trustworthy, and the information was also given to my husband." (Clark, 2014) |
|  | Targeted awareness initiatives | Those in the child welfare system were described as having few supports to learn anything about their own bodies, let alone to have anyone following up whether they receive the HPV vaccine within school-based programs. (Henderson, 2018) |
|  |  | Others could appreciate that it might be difficult from a school's perspective to educate in detail all families about HPV, but these speakers proposed that school-based programs alone were not entirely effective, and instead require outreach to older generations as well, “to get the word out.” (Henderson, 2018) |
|  |  | The importance of verbal forms of education was among the strongest themes, underscoring that prevention without relationships would be unlikely to improve HPV vaccine uptake or related health outcomes. (Henderson, 2018) |
|  |  | "For us to get out there and reach these people, we have to know what we are talking about…We need to be educated on it before we can take it and present it to people in our communities….” (Schmidt-Grimminger, 2013) |
|  |  | The groups mentioned education through venues such as health fairs, radio announcements, and posters. The tribal and IHS healthcare providers discussed the importance of culturally appropriate education and outreach. For example, the tribal healthcare providers noted that some materials should be in their native language (Lakota) in order to successfully conduct outreach to elders of the community who are important opinion leaders in their community. Similarly, the IHS providers noted that they could provide more educational materials in the clinic but stated that it would be better if they were culturally specific to the Northern Plain. (Schmidt-Grimminger, 2013) |
|  | Re-consideration of current practices | "It would be better if it were the same as the rest of the vaccines they give to the newborns, at three months, six months, four months. I’d prefer it more if it was like that, so that it would be more effective, just like the other vaccines. And so that there would be a way to keep track, like the other [vaccine record] cards. It would be the same and there it could integrate into that group of vaccines." (Clark, 2014) |
|  |  | Many women expressed the idea that 9 years old was too young to get a vaccine that protected against a sexually transmitted disease because the child was still a few years away from having sex, and it was at that later point that the decision should be made about vaccination or not. (Bowen, 2014) |
|  |  | Some women pointed out that by the older age, it might be too late, and that if girls had to bring up a vaccine when they were considering having sex, it would operate the same as birth control in that if a child brings up birth control, it means that she is having sex and needs it. (Bowen, 2014) |
|  |  | Recognizing a complex sequence of risks that may heighten vulnerability, the participant ensured that her daughter received the HPV vaccine at school, wishing only that it had been available sooner. (Henderson, 2018) |
